# Longitudinal change in castration-resistant prostate cancer biomarker AST/ALT ratio reflects tumor progression

**DOI:** 10.1038/s41598-023-42711-z

**Published:** 2023-09-15

**Authors:** Yozo Mitsui, Fumito Yamabe, Shunsuke Hori, Masato Uetani, Hiroshi Aoki, Kei Sakurabayashi, Mizuho Okawa, Hideyuki Kobayashi, Koichi Nagao, Koichi Nakajima

**Affiliations:** Department of Urology, Faculty of Medicine, Toho University, Tokyo, 143-8540 Japan

**Keywords:** Cancer, Urological cancer, Prostate cancer

## Abstract

We investigated whether aspartate transaminase (AST)-to-alanine aminotransferase (ALT) ratio and its change during the course of treatment in castration-resistant prostate cancer (CRPC) patients is associated with tumor condition and lethality. Clinical data from 130 CRPC patients were retrospectively evaluated. AST/ALT ratios at the time of prostate cancer (PC) diagnosis, androgen deprivation therapy (ADT), CRPC diagnosis, and the final follow-up examination after CRPC treatment were calculated for each. The prognostic capabilities of the AST/ALT ratio for overall survival (OS) were analyzed by use of the Kaplan–Meier method and Cox hazard models. The median AST/ALT ratio at PC diagnosis was 1.517 and the optimal value predicting lethality defined by the receiver operating curve was 1.467. The AST/ALT ratio decreased once during ADT and then elevated in a stepwise manner with cancer progression. In surviving patients, the median AST/ALT ratio at the time of PC diagnosis was 1.423, which did not change longitudinally, whereas that in patients later deceased was significantly higher (1.620) and further elevated after CRPC diagnosis. Kaplan–Meier curves indicated significantly worse OS in patients with an AST/ALT ratio ≥ 1.467, which was confirmed by multivariate analysis. These findings indicate AST/ALT ratio as a prognostic biomarker for CRPC with longitudinal changes reflecting tumor progression.

## Introduction

Prostate cancer (PC) is the most common type of cancer occurring in men worldwide. Most newly diagnosed patients are in an early stage and have an excellent outcome with local treatment. In contrast, many with recurrent or metastatic PC are given systemic treatment with androgen deprivation therapy (ADT) because of proliferation in an androgen-dependent manner. Unfortunately, the anti-tumor effect of ADT on hormone-sensitive PC (HSPC) is only temporary, and most patients develop lethal castration-resistant prostate cancer (CRPC) within 6 months to several years^1,2^. Over the previous decade, several new CRPC agents have become available, such as next-generation hormonal agents, paclitaxel, and radium-223. However, even with multiple lines of treatment with these agents, the outcomes of PC patients after acquiring castration resistance are comparatively unsatisfactory, with overall survival (OS) of metastatic CRPC (mCRPC) cases less than 3 years and survival of non-metastatic CRPC (nmCRPC) cases only 10 months longer^3,4^.

In recent years, treatment strategies for CRPC have changed dramatically, with delay of progression to CRPC as much as possible considered to be a key point for improving prognosis of PC patients^5,6^. For example, the mainstay treatment for metastatic HSPC (mHSPC) has become upfront therapy, which combines conventional ADT and agents used for CRPC. This change in treatment strategy is based on results of several large phase III trials showing that a combination of ADT with androgen receptor axis-targeted therapy (ARAT) or docetaxel (DTX) significantly improved prognosis as compared with ADT alone in patients with mHSPC^7–11^. In addition, mHSPC has been shown to have a pronounced genomic heterogeneity among patients, who accumulate various genetic mutations associated with poor prognosis and treatment resistance during the process of acquiring castration resistance^12–15^. Therefore, by combining ADT with both ARAT and DTX, which have different mechanisms of action against cancer, an intense combination known as triplet therapy that inhibits as much as possible the pathway that acquires castration resistance from an early stage is becoming popular^16,17^. We believe that factors that can accurately reflect tumor progression and predict patient prognosis will play important roles in selection of such intensive combination therapy for HSPC cases.

Aspartate transaminase (AST) and alanine aminotransferase (ALT) are well-known blood biomarkers that reflect liver damage. AST to ALT ratio (AST/ALT ratio), also termed De Ritis ratio, was first proposed in a study of hepatitis etiology and is now commonly used to distinguish the cause of liver disease. It is also considered that AST/ALT ratio may be an effective biomarker for diseases other than liver disease. For example, elevated AST/ALT ratio has been reported to be correlated with cancer development and poor prognosis in various types of malignancies^18–20^. Regarding PC, AST/ALT ratio at the time of prostatic biopsy was found significantly elevated in PC as compared to benign prostatic hyperplasia patients^21^. Furthermore, several interesting studies have shown that higher AST/ALT ratio at each stage may be significantly associated with prostate specific antigen (PSA) recurrence after radical surgery or radiotherapy, as well as prognosis after diagnosis of CRPC^22–24^.

It is speculated that AST/ALT ratio at each stage reflects biological malignancy of the tumor at each PC stage and longitudinally changes with cancer progression. The present study was conducted to investigate AST/ALT ratio and its changes during the course of treatment from time of PC diagnosis in CRPC patients in correlation with tumor progression and prognosis.

## Results

### Longitudinal assessment of AST/ALT ratio

**Figure 1:**
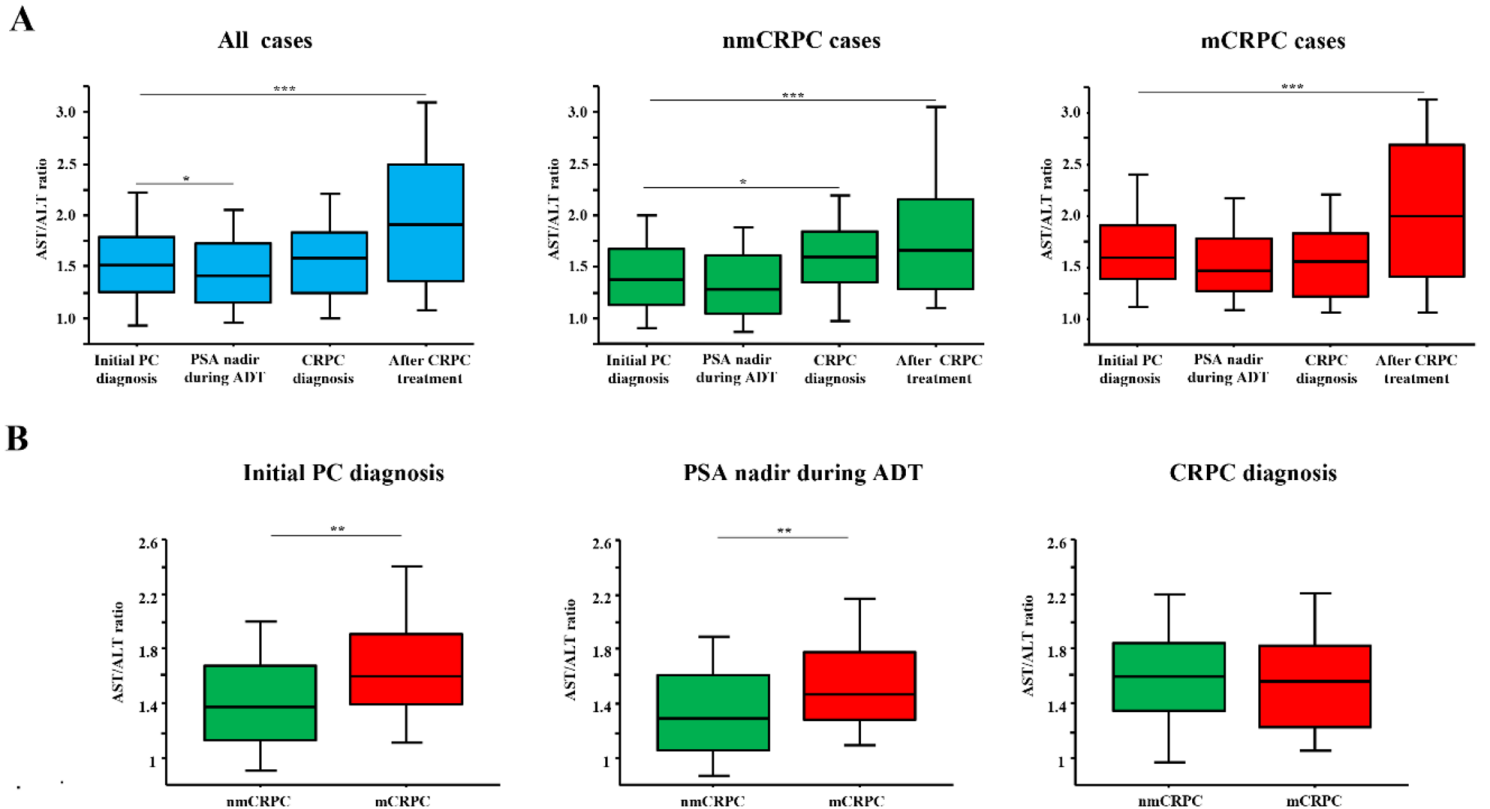
Longitudinal changes in AST/ALT ratio during course of treatment in patients with castration-resistant prostate cancer. (**A**) Longitudinal changes in AST/ALT ratio over course of treatment in total cohort, and nmCRPC and mCRPC groups. The ratio decreased once during ADT and then elevated in a stepwise manner with cancer progression. *p < 0.05, ***p < 0.001. (**B**) Comparison of AST/ALT ratios for nmCRPC and mCRPC groups at each stage. Metastatic CRPC patients had a significantly higher AST/ALT ratio at the time of PC diagnosis as compared to those without metastases, while there was no significant difference after CRPC diagnosis. **p < 0.01.

Longitudinal changes in AST/ALT ratio over the course of treatment in the total cohort, and nmCRPC and mCRPC cases are shown in Fig. 1A. The median AST/ALT ratio at PC diagnosis for the entire cohort was 1.517 (1.260–1.794), which then significantly decreased to 1.419 (1.151–1.737) at the PSA nadir during subsequent ADT treatment (p = 0.017). At CRPC diagnosis, AST/ALT ratio increased to 1.583 (1.247–1.836), nearly the same as at PC diagnosis. Subsequently, it increased to 1.913 (1.360–2.500) at the final observation period after CRPC treatment, significantly higher than at PC diagnosis (p < 0.001). Although the reduction in AST/ALT ratio during ADT treatment did not reach statistical significance (p = 0.082), a similar trend was observed in nmCRPC cases, notably the AST/ALT ratio at CRPC diagnosis was significantly higher than at PC diagnosis (p = 0.011). In mCRPC cases, AST/ALT ratio changes were slight until progression to CRPC, then increased significantly after CRPC treatment (p < 0.001). A comparison of AST/ALT ratios between nmCRPC and mCRPC at each stage is shown in Fig. 1B. mCRPC cases had a significantly higher AST/ALT ratio than nmCRPC cases at PC diagnosis (1.375 vs. 1.600, p = 0.008) and during ADT treatment (1.286 vs. 1.462, p = 0.008). Notably, at CRPC diagnosis, AST/ALT ratio in nmCRPC cases increased to 1.600 (1.342–1.857), no longer significantly different than mCRPC at 1.563 (1.221–1.832).

**Figure 2:**
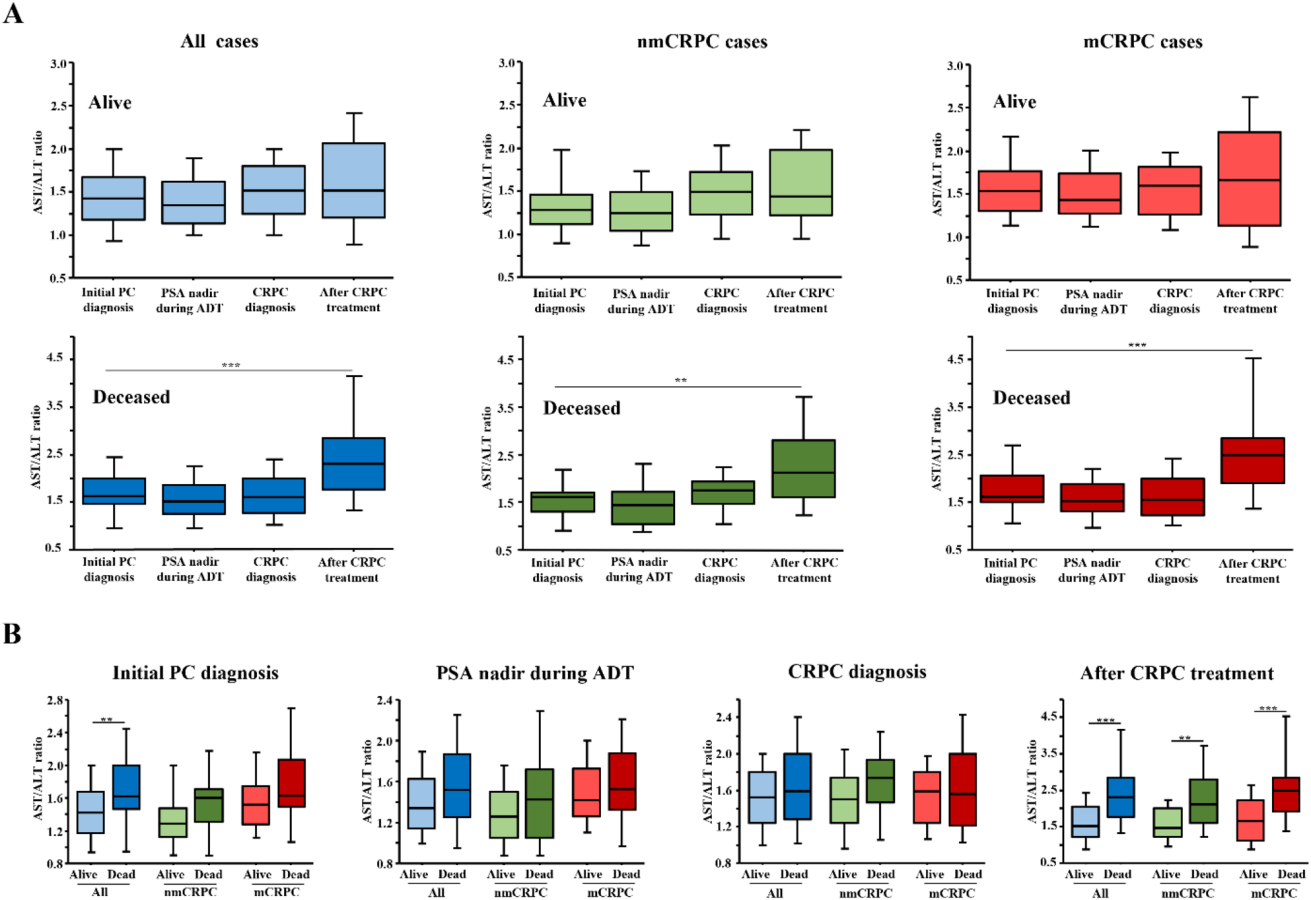
Longitudinal changes in AST/ALT ratio in castration-resistant prostate cancer patients classified based on survival. (**A**) Longitudinal changes in AST/ALT ratio during treatment course according to patient survival in the total cohort, and nmCRPC and mCRPC groups. During the follow-up period, the AST/ALT ratio did not show a significant longitudinal change after PC diagnosis in surviving patients, whereas it was increased after CRPC diagnosis in deceased patients. **p < 0.01, ***p < 0.001. (**B**) Comparison of AST/ALT ratio differences between live and deceased patients at each stage in the three cohorts. At the time of PC diagnosis, the AST/ALT ratio in the total cohort was significantly greater for deceased patients. After CRPC treatment, AST/ALT ratio was also significantly higher for deceased patients in each of the three cohorts. **p < 0.01.

Next, longitudinal changes in AST/ALT ratio during the treatment course according to patient survival were assessed. Sixty (46.2%) of the 130 patients died during the follow-up period, all from cancer-specific causes. The AST/ALT ratios of all patients who survived during the observation period did not change significantly from PC diagnosis to after CRPC treatment (Fig. 2A). In contrast, that ratio in patients who died was significantly elevated at the final observation after CRPC treatment as compared to PC diagnosis in the total cohort, and nmCRPC and mCRPC cases (p < 0.001, p = 0.008, p < 0.001, respectively). AST/ALT ratio differences between live and dead patients at each stage in the three cohorts were compared (Fig. 2B). Interestingly, at PC diagnosis, the AST/ALT ratio for patients who were later deceased cases as compared to the total cohort was significantly greater [1.620 (1.468–2.000) vs. 1.423 (1.171–1.682), p = 0.004). During ADT treatment and at CRPC diagnosis, AST/ALT ratios did not differ significantly between surviving and deceased patients in any cohort. On the other hand, following CRPC treatment, the AST/ALT ratio was significantly elevated in deceased as compared to surviving patients in the entire cohort, and nmCRPC and mCRPC groups (p < 0.001, p = 0.007, p < 0.001, respectively).

### Longitudinal assessment of AST and ALT values

**Figure 3:**
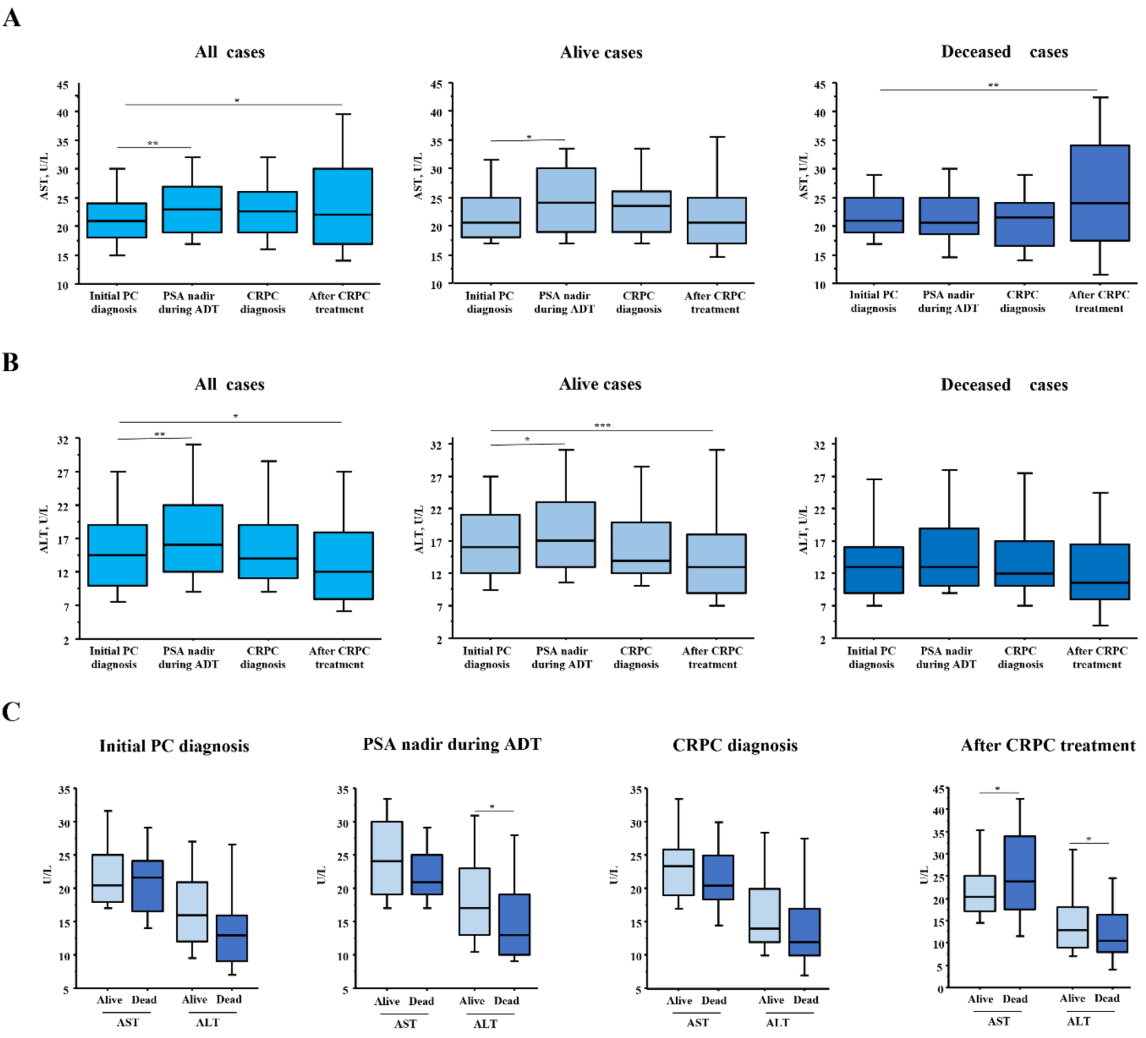
Longitudinal changes in AST or ALT during course of treatment in patients with castration-resistant prostate cancer. (**A**) Longitudinal changes in serum AST levels according to patient survival. AST level increased gradually after PC diagnosis and was significantly higher after CRPC treatment than at the time of PC diagnosis. This finding was noted in all including deceased cases. *p < 0.05, ** p < 0.01. (**B**) Longitudinal changes in serum ALT levels according to patient survival. A longitudinal decreasing trend in ALT during treatment was observed. *p < 0.05, **p < 0.01, ***p < 0.001. (**C**) Comparison of differences in AST and ALT values between living and deceased patients at each stage. Following CRPC treatment, deceased patients had significantly higher AST and lower ALT values as compared with those who survived. *p < 0.05.

Longitudinal changes in serum AST and ALT levels were also evaluated. AST levels gradually increased after PC diagnosis and were significantly higher after CRPC treatment than at PC diagnosis in the total cohort (21 vs. 22, p = 0.047) (Fig. 3A). A similar trend was observed for deceased patients, with AST significantly elevated after CRPC treatment as compared to time of PC diagnosis (21 vs. 24, respectively, p = 0.001). However, in surviving patients, AST level increased once during ADT treatment but then decreased to the same level as at PC diagnosis. Additionally, ALT level in the total cohort increased once after PC diagnosis and then significantly declined after CRPC treatment (15 vs. 12, p = 0.039). (Fig. 3B). Although the difference was not statistically significant for deceased cases, a longitudinal decreasing trend in ALT during treatment was observed in both surviving and deceased patients (Fig. 3B). Finally, comparisons of AST and ALT in survivors and deceased patients at each stage showed that patients who died had significantly higher AST and lower ALT levels than survivors after CRPC treatment (Fig. 3C).

### Evaluation of AST/ALT ratio as prognostic predictor

**Figure 4:**
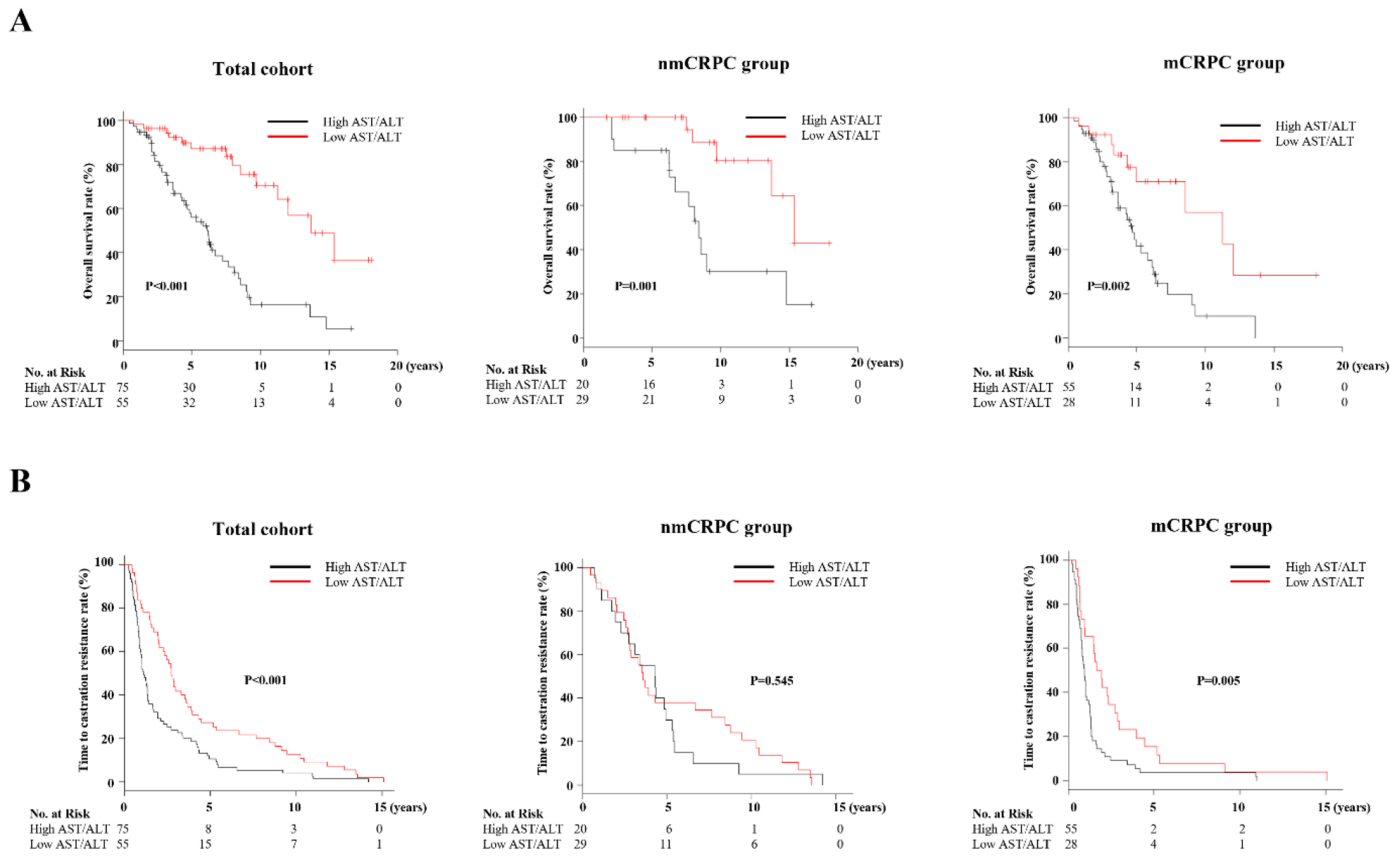
Kaplan–Meier analysis of overall survival after prostate cancer diagnosis, and time to castration resistance (TTCR) following androgen deprivation therapy based on AST/ALT ratio. (**A**) Kaplan–Meier curve analysis showed that patients with a high AST/ALT ratio had significantly worse OS than those with a low ratio in each of the three groups. (**B**) A significantly shorter TTCR was observed in patients with a high AST/ALT ratio as compared to those with a low AST/ALT ratio in the total cohort and mCRPC group.

Findings of higher AST/ALT ratio at PC diagnosis for deceased cases led to speculation regarding potential for predicting prognosis of CRPC patients. Optimal cutoff values for AST/ALT ratio at PC diagnosis for lethality prediction in CRPC patients examined by ROC curve analysis using Youden’s index revealed a value of 1.467 (AUC 0.646, sensitivity 0.767, specificity 0.586) (Supplemental Fig. 1). Using that as a cutoff level, patients were divided into low (< 1.467, n = 55) and high (≥ 1.467, n = 75) AST/ALT ratio groups, with their clinicopathological characteristics shown in Table 1. Briefly, the high AST/ALT ratio group was significantly older, had lower hemoglobin and albumin levels, higher regional lymph node and distant metastasis rates, greater rate of high tumor volume, and shorter TTCR. In addition, the high AST/ALT ratio group consistently had a significantly higher AST/ALT ratio at subsequent stages. Kaplan–Meier curve analysis showed that the high AST/AT ratio group had significantly worse OS than the low AST/ALT group in the total cohort (median 50.4 vs. 85.2 months, p < 0.001), and nmCRPC (median 86.3 vs. 110.1 months, p = 0.001) and mCRPC (median 38.4 vs 54.0 months, p = 0.001, p = 0.002) groups (Fig. 4A). Furthermore, significantly shorter TTCR was observed in patients with a high AST/ALT ratio in the total cohort (median 14.2 vs. 33.4 months, p < 0.001) and mCRPC group (median 11.2 vs. 21.6 months, p = 0.005) (Fig. 4B).

**Table 1:** Clinicopathological characteristics for entire cohort and subgroups according to AST/ALT ratio.

Characteristics	Total cohort	Low AST/ALT ratio	High AST/ALT ratio	p value
	n = 130	n = 55	n = 75	
Age at PC diagnosis, years	73.4 ± 8.6	71.6 ± 8.6	74.8 ± 8.3	0.037
Body mass index, kg/m^2^	23.0 ± 3.8	23.1 ± 3.1	22.9 ± 4.3	0.773
ECOG PS at PC diagnosis				0.333
0, 1	101 (77.7)	45 (81.8)	56 (74.7)	
≥ 2	29 (22.3)	10 (19.2)	19 (25.3)	
Serum markers at PC diagnosis				
PSA level, ng/mL	99.1 (25.4–489.7)	82.2 (24.5–307.0)	106.5 (25.6–520.6)	0.371
Hemoglobin, g/dL	13.2 ± 1.9	13.8 ± 1.7	12.2 ± 1.7	0.005
White blood cells, × 10^9^/L	6.0 ± 1.8	5.9 ± 1.6	6.0 ± 1.9	0.644
Lactate dehydrogenase, U/L	215 (184–256)	214 (185–256)	217 (181–257)	0.976
Alkaline phosphatase, U/L	242 (184–381)	231 (184–310)	276 (182–475)	0.030
AST, U/L	21 (18–24)	22 (18–26)	20 (17–24)	0.112
ALT, U/L	15 (10–19)	19 (16–27)	11 (9–15)	< 0.001
Total protein, g/dL	7.4 ± 0.5	7.5 ± 0.5	7.4 ± 0.5	0.133
Albumin, g/dL	4.1 ± 0.6	4.2 ± 0.6	3.9 ± 0.5	0.001
CRP, mg/L	0.1 (0–0.4)	0.1 (0–0.3)	0.1 (0.1–0.6)	0.073
AST/ALT ratio				
PC diagnosis	1.52 (1.26–1.79)	1.19 (0.96–1.32)	1.75 (1.60–2.10)	< 0.001
PSA nadir during ADT	1.42 (1.15–1.74)	1.22 (1.05–1.42)	1.60 (1.38–1.89)	< 0.001
CRPC diagnosis	1.58 (1.25–1.84)	1.41 (1.06–1.71)	1.70 (1.42–1.91)	0.001
After CRPC treatment	1.91 (1.36–2.50)	1.63 (1.23–2.17)	2.11 (1.61–2.71)	0.001
Clinical T stage				0.127
≤ T3	111 (85.4)	50 (90.9)	61 (71.8)	
T4	19 (14.6)	5 (10.1)	14 (28.2)	
Gleason score				0.265
≥ 9	57 (43.8)	21 (38.2)	36 (48.0)	
Regional lymph node metastasis	48 (36.9)	14 (25.5)	34 (45.3)	0.020
Distant metastasis at PC/CRPC diagnosis	81 (62.3)	26 (47.3)	55 (73.3)	0.003
Distant metastatic site at PC diagnosis				
Bone (total)	78 (60.0)	25 (45.5)	53 (70.7)	0.004
Any viscera (lung, liver, lymph node)	20 (15.4)	4 (7.3)	16 (21.3)	0.028
Tumor burden according to CHAARTED				0.001
High	59 (45.4)	15 (27.3)	44 (58.7)	
Low	22 (16.9)	11 (20.0)	11 (14.7)	
Non-metastasis	49 (37.7)	29 (52.7)	20 (14.6)	
Local therapy				0.375
None	117 (90.0)	48 (87.3)	69 (92.0)	
Yes	13 (10.0)	7 (12.7)	6 (8.0)	
Time to castration resistance, months	19.7 (9.9–46.9)	33.1 (17.7–64.1)	14.2 (9.0–34.1)	< 0.001
First-line treatment for CRPC				0.013
ARAT	73 (56.2)	38 (69.1)	35 (46.7)	
First-generation antiandrogen	46 (35.3)	11 (20.0)	35 (46.7)	
Docetaxel	10 (7.7)	5 (10.1)	5 (6.6)	
Radium-223	1 (0.8)	1 (1.8)	0 (0)	
Died during observation period	60 (46.2)	14 (25.5)	46 (61.3)	< 0.001

Data are presented as median (interquartile range), mean ± standard deviation, or number (percentage).

*PC* prostate cancer, *ECOG PS* Eastern Cooperative Oncology Group Performance Status scale, *PSA* prostate-specific antigen, *AST* aspartate aminotransferase, *ALT* alanine aminotransferase, *CRP* c-reacted protein, *ADT* androgen deprivation therapy, *CRPC* castration-resistant prostate cancer, *nmCRPC* non-metastatic CRPC, *mCRPC* metastatic CRPC, *ARAT* androgen receptor axis-targeted treatment.

To further assess the prognostic value of AST/ALT ratio, uni- and multivariate Cox analyses of OS and TTCR incorporating patient characteristics at PC diagnosis were performed. Continuous variables other than AST/ALT ratio were used to classify into two groups based on median or mean values. As shown in Table 2, AST/ALT ratio, age, BMI, PS, hemoglobin, white blood cell count, ALT level, T stage, Gleason score (GS), regional lymph node metastasis, and distant metastasis were significantly independent predictors for OS in the total cohort. When applied to a multivariate model, BMI, PS, AST/ALT ratio, GS, regional lymph node involvement, and distant metastasis were found to be significantly independent predictors of OS, among which AST/ALT ratio was most relevant (HR 4.639, 95% CI 2.04–10.6, p < 0.001). AST/ALT ratio was also a candidate factor for a significant association with TTCR in univariate but not multivariate analysis. Similarly, AST/ALT ratio was identified as a strong factor associated with OS by multivariate analysis in both the nmCRPC (HR 3.461, 95% CI 1.16–10.3, p = 0.026) and mCRPC (HR 2.446, 95% CI 1.10–5.43, p = 0.028) groups (Table 3). Although univariate analysis showed AST/ALT ratio as a candidate factor for TTCR in mCRPC cases, it was not a significant factor in multivariate analysis (Table 3).

**Table 2:** Uni- and multivariate Cox proportional hazards analysis findings for overall survival rate and time to castration resistance in 130 CRPC cases.

Characteristics	Overall survival				Time to castration resistance			
	Univariate		Multivariate		Univariate		Multivariate	
	HR (95% CI)	p value	HR (95% CI)	p value	HR (95% CI)	p value	HR (95% CI)	p value
Age (≥ 73.5 years)	2.241 (1.30–3.86)	0.004	1.607 (0.85–3.02)	0.141	1.786 (1.24–2.57)	0.002	1.731 (1.15–2.60)	0.008
Body mass index (≥ 23.0 kg/m^2^)	0.441 (0.25–0.78)	0.005	0.409 (0.21–0.82)	0.011	0.972 (0.69–1.38)	0.875	–	–
ECOG PS (≥ 1)	2.162 (1.26–3.71)	0.005	2.051 (1.06–3.96)	0.032	0.915 (0.60–1.39)	0.675	–	–
Hemoglobin (≥ 13.2 g/dL)	0.515 (0.31–0.86)	0.011	1.308 (0.68–2.51)	0.418	0.532 (0.37–0.76)	0.001	0.964 (0.62–1.51)	0.874
White blood cells (≥ 6000 × 10^9^/L)	1.722 (1.03–2.89)	0.040	1.417 (0.82–2.44)	0.209	1.302 (0.91–1.86)	0.148	–	–
Lactate dehydrogenase (≥ 215 U/L)	1.206 (0.71–2.04)	0.484	–	–	0.808 (0.57–1.16)	0.244	–	–
Alkaline phosphatase (≥ 242 U/L)	1.670 (1.06–2.80)	0.051	–	–	1.368 (0.96–1.95)	0.083	–	–
Total protein (≥ 7.4 g/dL)	1.082 (0.64–1.82)	0.765	–	–	0.939 (0.66–1.33)	0.723	–	–
Albumin (≥ 4.1 g/dL)	0.616 (0.37–1.03)	0.067	–	–	0.611 (0.43–0.87)	0.006	0.744 (0.49–1.13)	0.169
AST (≥ 21 U)	1.280 (0.76–2.16)	0.355	–	–	1.143 (0.80–1.62)	0.457	–	–
ALT (≥ 15 U)	0.521 (0.37–0.88)	0.014	1.880 (0.89–3.96)	0.097	0.835 (0.59–1.18)	0.311	–	–
CRP (≥ 0.2 mg/dL)	1.254 (0.75–2.09)	0.384	–	–	1.384 (0.97–1.98)	0.074	–	–
PSA level (≥ 99.1 ng/mL)	1.511 (0.90–2.55)	0.122	–	–	1.468 (1.03–2.09)	0.034	1.223 (0.84–1.79)	0.300
AST/ALT ratio (≥ 1.467)	4.300 (2.34–7.91)	< 0.001	4.639 (2.04–10.6)	< 0.001	1.816 (1.27–2.59)	0.001	1.211 (0.81–1.82)	0.355
Clinical T stage (T4)	2.156 (1.10–4.23)	0.025	1.089 (0.50–2.38)	0.831	2.148 (1.31–3.53)	0.003	1.619 (0.90–2.92)	0.109
Gleason score (≥ 9)	2.542 (1.48–4.37)	< 0.001	1.983 (1.11–3.54)	0.020	1.779 (1.24–2.57)	0.002	1.794 (1.21–2.66)	0.004
Regional lymph node metastasis	3.692 (2.10–6.50)	< 0.001	3.470 (1.69–7.13)	< 0.001	3.339 (2.25–4.95)	< 0.001	2.351 (1.48–3.73)	< 0.001
Distant metastasis	4.155 (2.26–7.63)	< 0.001	2.114 (1.05–4.27)	0.037	2.607 (1.80–3.77)	< 0.001	3.158 (1.36–3.16)	< 0.001

*HR* hazard ratio, *ECOG PS* Eastern Cooperative Oncology Group Performance Status scale, *AST* aspartate aminotransferase, *ALT* alanine aminotransferase, *CRP* c-reacted protein, *PSA* prostate-specific antigen, *CRPC* castration-resistant prostate cancer, *AA* antiandrogen.

**Table 3:** Uni- and multivariate Cox proportional hazards analysis findings for overall survival rate and time to castration resistance in metastatic and non-metastatic CRPC cases.

Characteristics	Overall survival				Time to castration resistance			
	Univariate		Multivariate		Univariate		Multivariate	
	HR (95% CI)	p value	HR (95% CI)	p value	HR (95% CI)	p value	HR (95% CI)	p value
Non-metastatic CRPC								
Age (≥ 73.5 years)	4.072 (1.35–12.3)	0.013	3.892 (1.16–13.1)	0.028	2.032 (1.01–3.84)	0.029	1.938 (0.99–3.81)	0.055
Body mass index (≥ 23.0 kg/m^2^)	0.476 (0.17–1.30)	0.148	–	–	1.646 (0.90–3.02)	0.108	–	–
ECOG PS (≥ 1)	3.037 (1.10–8.42)	0.033	3.398 (1.12–10.3)	0.031	0.646 (0.30–1.40)	0.267	–	–
Hemoglobin (≥ 13.2 g/dL)	0.837 (0.27–2.60)	0.758	–	–	0.353 (0.18–0.71)	0.004	0.249 (0.11–0.56)	< 0.001
White blood cells (≥ 6000 × 10^9^/L)	1.478 (0.55–3.95)	0.436	–	–	1.983 (1.01–3.66)	0.028	2.925 (1.49–5.76)	0.002
Lactate dehydrogenase (≥ 215 U/L)	1.149 (0.42–3.18)	0.789	–	–	0.558 (0.31–1.01)	0.055	–	–
Alkaline phosphatase (≥ 242 U/L)	0.481 (0.14–1.69)	0.253	–	–	0.975 (0.52–1.81)	0.937	–	–
Total protein (≥ 7.4 g/dL)	1.157 (0.42–3.20)	0.779	–	–	1.061 (0.60–1.88)	0.840	–	–
Albumin (≥ 4.1 g/dL)	0.818 (0.30–2.23)	0.695	–	–	0.672 (0.37–1.21)	0.187	–	–
AST (≥ 21 IU)	2.378 (0.80–7.06)	0.119	–	–	1.092 (0.61–1.97)	0.771	–	–
ALT (≥ 15 IU)	0.433 (0.17–1.13)	0.088	–	–	0.831 (0.46–1.52)	0.546	–	–
CRP (≥ 0.2 mg/dL)	0.966 (0.37–2.55)	0.945	–	–	1.534 (0.85–2.77)	0.156	–	–
PSA levels (≥ 99.1 ng/mL)	0.847 (0.23–3.07)	0.800	–	–	1.586 (0.85–2.95)	0.145	–	–
AST/ALT ratio (≥ 1.467)	4.738 (1.65–13.6)	0.004	3.461 (1.16–10.3)	0.026	1.201 (0.67–2.17)	0.546	–	–
Clinical T stage (T4)	1.028 (0.13–7.96)	0.979	–	–	1.961 (0.77–5.03)	0.161	–	–
Gleason score (≥ 9)	1.040 (0.38–2.85)	0.939	–	–	1.847 (1.01–3.39)	0.047	1.600 (0.86–2.99)	0.141
Regional lymph node metastasis	2.441 (0.65–9.16)	0.186	–	–	2.894 (1.31–6.40)	0.009	4.915 (2.03–11.9)	< 0.001
Local therapy	3.039 (0.84–11.0)	0.091	–	–	0.868 (0.45–1.66)	0.669	–	–
Metastatic CRPC								
Age (≥ 73.5 years)	1.465 (0.80–2.70)	0.219	–	–	1.465 (0.80–2.70)	0.219	–	–
Body mass index (≥ 23.0 kg/m^2)^	0.524 (0.26–1.04)	0.066	–	–	0.997 (0.63–1.57)	0.990	–	–
ECOG PS (≥ 1)	1.695 (0.89–3.22)	0.107	–	–	0.831 (0.50–1.38)	0.477	–	–
Hemoglobin (≥ 13.2 g/dL)	0.592 (0.32–1.08)	0.089	–	–	0.772 (0.50–1.20)	0.249	–	–
White blood cells (≥ 6000 × 10^9^/L)	1.489 (0.81–2.74)	0.202	–	–	0.946 (0.61–1.48)	0.808	–	–
Lactate dehydrogenase (≥ 215 U/L)	1.141 (0.62–2.10)	0.673	–	–	1.077 (069–1.69)	0.747	–	–
Alkaline phosphatase (≥ 242 U/L)	1.628 (0.85–3.13)	0.144	–	–	1.083 (0.69–1.70)	0.728	–	–
Total protein (≥ 7.4 g/dL)	1.059 (0.58–1.94	0.852	–	–	0.854 (0.54–1.35)	0.496	–	–
Albumin (≥ 4.1 g/dL)	0.621 (0.34–1.15)	0.128	–	–	0.662 (0.93–2.26)	0.104	–	–
AST (≥ 21 IU)	1.143 (0.62–2.12)	0.672	–	–	1.446 (0.80–2.73)	0.219	–	–
ALT (≥ 15 IU)	0.779 (0.41–1.47)	0.441	–	–	1.054 (0.67–1.66)	0.819	–	–
CRP (≥ 0.2 mg/dL)	1.240 (0.67–2.28)	0.491	–	–	1.190 (0.76–1.86)	0.445	–	**–**
PSA level (≥ 99.1 ng/mL)	0.935 (0.50–1.75)	0.800	–	–	0.959 (0.61–1.50)	0.586	–	**–**
AST/ALT ratio (≥ 1.467)	3.043 (1.43–6.47)	0.004	2.446 (1.10–5.43)	0.028	1.968 (1.21–3.20)	0.006	1.491 (0.88–2.54)	0.140
Clinical T stage (T4)	1.864 (0.90–3.86)	0.094	–	–	2.001 (1.11–3.62)	0.022	1.073 (0.56–2.08)	0.833
Gleason score (≥ 9)	2.349 (1.24–4.44)	0.009	2.053 (1.08–3.92)	0.029	1.789 (1.13–2.84)	0.014	1.406 (0.87–2.28)	0.169
Regional lymph node metastasis	2.688 (1.39–5.19)	0.003	1.841 (0.93–3.66)	0.082	2.489 (1.55–3.99)	< 0.001	2.048 (1.21–3.47)	0.008
Bone metastasis	4.442 (0.60–32.7)	0.143	–	–	2.000 (0.72–5.56)	0.184	–	–
Any viscera metastasis	0.611 (0.31–1.20)	0.151	–	–	0.656 (0.39–1.10)	0.107	–	–
Tumor burden (high)	1.841 (0.88–3.87)	0.107	–	–	1.547 (0.94–2.55)	0.088	–	–

*HR* hazard ratio, *CRPC* castration-resistant prostate cancer, *ECOG PS* Eastern Cooperative Oncology Group Performance Status scale, *AST* aspartate aminotransferase, *ALT* alanine aminotransferase, *CRP* c-reacted protein, *PSA* prostate-specific antigen, *AA* antiandrogen.

**Figure 5:**
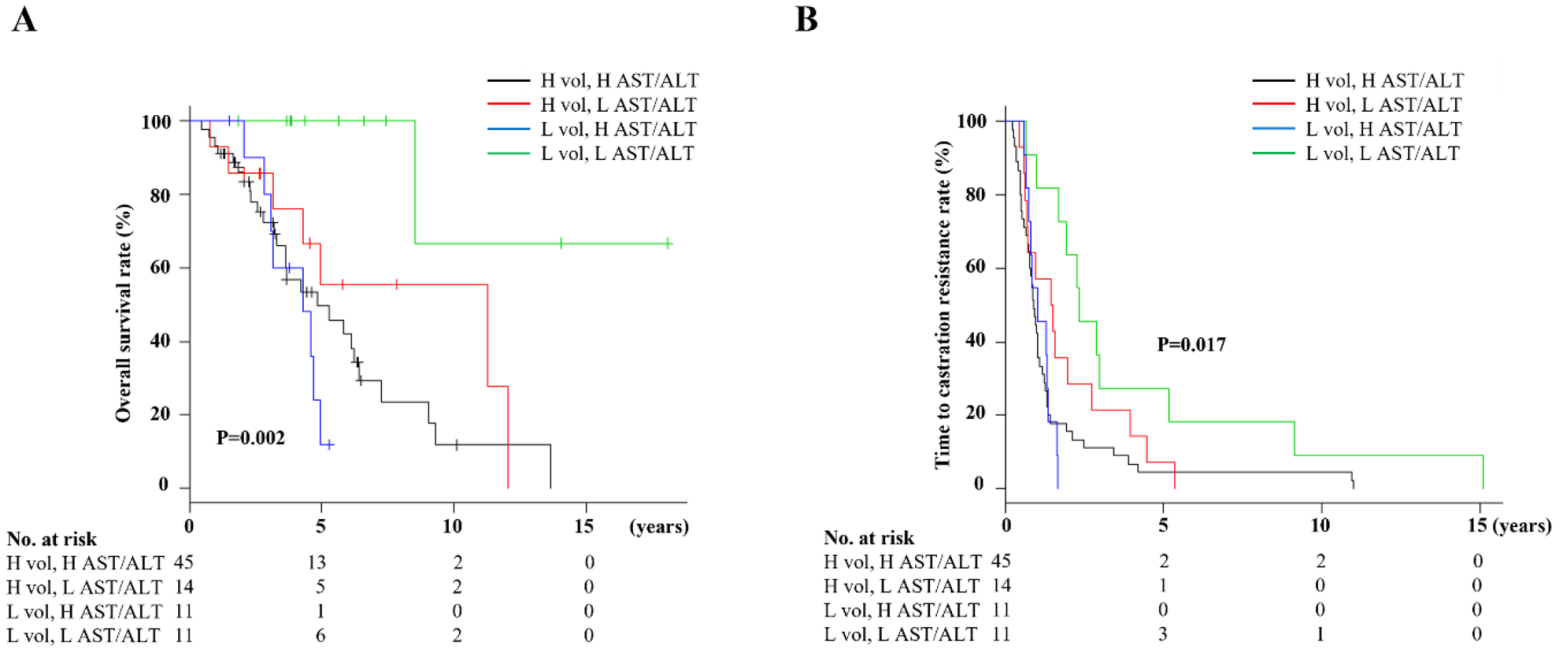
Kaplan–Meier analysis of overall survival and time to castration resistance in 81 mCRPC patients according to tumor volume and AST/ALT ratio. (**A**,**B**) The accuracy of predicting OS and TTCR in patients with advanced mCRPC from mHSPC classified by tumor burden was improved by subdividing by AST/ALT ratio. Low-volume patients with a high AST/ALT ratio had a similar poor prognosis and short TTCR as compared to high-volume patients with a high AST/ALT ratio.

Since AST/ALT ratio was found to be associated with tumor burden (Table 1), the prognosis of 81 mCRPC patients who progressed from mHSPC (high tumor volume n = 59, low tumor volume n = 22) was examined by combining tumor burden and AST/ALT ratio. The prediction accuracy of OS and TTCR in mCRPC cases classified by tumor burden was improved by combining with AST/ALT ratio (Fig. 5A,B). Notably, among patients classified as low tumor volume, those with higher AST/ALT ratios for both OS and TTCR were significantly worse as compared to those with lower AST/ALT ratios. Nonetheless, no differences other than AST/ALT ratios were found between the two groups.

## Discussion

This is the first known investigation of longitudinal changes in AST/ALT ratio in CRPC patients during treatment, with several noteworthy findings obtained. First, the AST/ALT ratio changed significantly from time of PC diagnosis during the course of treatment. Specifically, the ratio decreased during ADT treatment, as the majority of cancer cells were suppressed, then subsequently increased with acquisition of castration resistance and continuing to increase after CRPC treatment. At the time of PC diagnosis, a higher AST/ALT ratio was significantly associated with factors representing cancer spread, such as distant metastasis to bone or visceral organs, regional lymph node metastasis, and high tumor volume. In addition, AST/ALT ratio after CRPC treatment in deceased patients, calculated at the time of terminal cancer progression, was significantly elevated as compared with that in surviving patients whose cancer progression was suppressed by continued treatment. These findings suggest that longitudinal changes in AST/ALT ratio reflect differences over time in response to treatment and progression of PC. Interestingly, Zhou et al. focused on the association of PC detection by prostatic needle biopsy with AST/ALT ratio and found a value in PC cases of 1.13, significantly higher as compared to benign prostatic hyperplasia cases (1.00)^21^. In a study that examined AST/ALT ratio as a prognostic factor in patients with radiation-recurrent PC who underwent a salvage radical prostatectomy, the median AST/ALT ratio was 1.33^22^, while Miyake et al. reported a median AST/ALT ratio of 1.35 in CRPC patients before treatment^24^. Although these findings were obtained at different hospitals, they suggest that AST/ALT ratio may change as PC progresses, and support our findings and speculation.

Second, AST/ALT ratio was significantly higher in the deceased cases at the time of PC diagnosis as compared to survivors. Based on this, AST/ALT ratio at PC diagnosis was examined, which showed that patients in all three cohorts with a high value (≥ 1.467) had worse OS than those with a low value (< 1.467), with those findings confirmed by uni- and multivariate analyses that incorporated various patient factors at time of PC diagnosis. AST/ALT ratio at PC diagnosis was also associated with TTCR in the total cohort and mCRPC groups, though found to be not significant in multivariate analysis. Of note, patients with a higher AST/ALT ratio at PC diagnosis consistently maintained a high value as compared those with a lower ratio as the stage progressed over time. There is pronounced genomic heterogeneity among patients at the time of PC diagnosis that develops into CRPC^12,13^. Therefore, PC cases with high malignancy due to several genomic abnormalities at the time of development are more likely to accumulate various genetic mutations associated with poor prognosis and subsequent treatment resistance. We also speculate that AST/ALT ratio may continue to change after PC diagnosis as a reflection of biological characteristics of the tumor including genomic variations. The present results showed that the AST/ALT ratio in patients with nmCRPC reached the same level as that in mCRPC patients after acquisition of castration resistance. As noted above, mHSPCs are often high-grade tumors with several genomic mutations occurring following the initial PC diagnosis, thus it is possible that the AST/ALT ratio was not significantly increased at the time of mCRPC diagnosis, with the tumor volume reduction effect of ADT another important factor. On the other hand, an nmCRPC, which progresses from non-meta HSPC with few genomic mutations, may become much more malignant than at the time of PC diagnosis during the process of acquiring castration resistance. The prognostic advantage of nmCRPC over mCRPC is only approximately 10 months^4^, thus CRPC is considered to be lethal regardless of metastasis. Elevated AST/ALT ratio after castration resistance in non-metastatic PC patients may reflect high malignancy in CRPC cases. Thus, we consider that AST/ALT ratio is a useful prognostic predictor for CRPC patients that reflects biological high malignancy from the time of PC diagnosis and then continues to change reflecting its alterations.

Finally, the accuracy of predicting OS and TTCR in mCRPC patients who progress from mHSPC, classified by tumor burden according to the CHAARTED criteria^25^, was confirmed to be improved by subdividing by AST/ALT ratio. It was surprising that the low volume patients with a high ratio showed the same poor prognosis and short TTCR as high volume patients. Akamatsu et al. constructed a unique predictive model combining GS, lactate dehydrogenase, and bone metastasis number, which showed poor prognosis for low volume patients^26^. Similarly, Shiota et al. successfully classified poor prognosis patients in a low volume group based on clinical factors such as hemoglobin and GS^27^. The current trend for mHSPC patients is upfront treatment, and its implementation and selection of therapeutic agents are often based on tumor burden classification. We believe that it is possible to accurately select mHSPC patients who should receive more intensive triplet therapy by considering tumor burden classification as well as high GS (≥ 9), regional lymph node metastasis, and AST/ALT ratio, identified in the present study as prognostic factors. Furthermore, using AST/ALT ratio, it may be possible to select patients including those with low volume who require active intervention with intensive therapy. It is also considered that AST/ALT ratio might be useful for treatment and follow-up of nmHSPC patients, since a higher value may predict poor prognosis.

Excessive proliferation of cancer cells requires a constant supply of fuel such as glucose and glutamine, a non-essential amino acid^28^. We speculate that AST/ALT ratio represents abnormalities in these pathways, and reflects the biological malignancy and prognosis of PC cases. Cancer cells exhibit enhanced anaerobic glycolysis, known as the Warburg effect, even in high-oxygen environments. Since anaerobic glycolysis is much less efficient at producing adenosine triphosphate (ATP) than aerobic glycolysis, these cells require large quantities of glucose. To increase ATP production, cancer cells enhance the malate-aspartate shuttle to facilitate transport of nicotinamide adenine dinucleotide generated by anaerobic glycolysis from cytosol to mitochondria. AST, also known as glutamate–oxaloacetate transaminases 1 and 2 (GOT1/2), is involved in driving that shuttle. Furthermore, the GOT 1/2-mediated malate-aspartate shuttle is associated with glutamine anaplerosis, a key mitochondrial metabolic pathway for cancer cell proliferation and survival^29^. GOT1/2, enzymes involved in glutamine metabolism, are both upregulated by K-Ras, known as an oncogene, in cancer cells^29^. It was found that GOT1 expression in cytoplasm was significantly higher in PC patients than healthy controls, and that the amount of glutamate metabolized from glutamine was increased in the order of normal, nmHSPC, and mHSPC patients^30,31^. Furthermore, both studies confirmed an association of GOT1 with cancer progression and acquisition of castration resistance, and that its knockdown suppresses cancer progression. Another report noted that ALT, unlike AST, is produced exclusively in the liver and largely unaffected by other factors such as cancer, and may decrease with liver aging^32^. The present findings that ALT was decreased and AST increased during long-term cancer treatment are consistent with those previous studies. Thus, increased AST/ALT ratio due to decreased or plateaued ALT and increased AST level may reflect activation of glucose and/or glutamine metabolic pathways in the tumor.

This study has several limitations, including its retrospective design and small number of patients at a single hospital. There may also be selection bias due to initial exclusion of patients with unavailable clinical data. Furthermore, it is possible that not all characteristics of PC patients were evaluated, as ADT-treated patients who did not progress to CRPC during the study period were not included. These limitations may also have influenced the lack of statistically significant difference for tumor volume, known to be an important prognostic factor in mHSPC patients. Exclusion of other candidate blood biomarkers including neutrophil–lymphocyte ratio is another limitation. Prospective studies with larger sized populations that overcome these limitations are required to externally validate and confirm the present results.

In conclusion, AST/ALT ratio was found to be a useful prognostic predictor for CRPC patients, as it longitudinally reflects both cancer progression, such as development of metastasis and increased tumor volume, and biological aggressiveness from the time of PC diagnosis. Consideration of AST/ALT ratio for selection of initial therapy for mHSPC patients as well as identification of nmHSPC patients with poor prognosis who progress to CRPC is recommended.

## Methods

### Patients and treatments

Of 172 CRPC patients treated at our hospital between September 1, 2009 and November 31, 2021, data from 159 (61 nmCRPC, 98 mCRPC cases) were retrospectively reviewed, after excluding 13 with ARAT and/or DTX in combination with initial ADT as upfront treatment at the time of HSPC diagnosis. New imaging techniques such as diffusion-weighted whole body imaging with background suppression were not available during the period of enrollment, thus hidden metastasis may have been present in some cases. To avoid the uncertain impact of such cases on the study results, one patient with mHSPC that progressed to nmCRPC and four with non-metastatic HSPC that progressed to mCRPC were excluded. Subsequently, 24 patients with a history of liver disease or no available longitudinal blood sampling data were excluded, thus 130 CRPC, including 81 with mCRPC that progressed from de novo mHSPC and 49 with nmCRPC that progressed from non-meta HSPC, were finally analyzed.

This study was conducted in compliance with the Declaration of Helsinki after being approved by the Ethics Committee of Toho University Omori Medical Center (no. M22169). Due to the retrospective nature of this study, it was decided by the Toho University Omori Medical Center Ethics Committee that the patient consent process could be omitted. However, information was posted on the hospital website regarding how to request exclusion.

### Assessments

Patient characteristics at PC diagnosis including age, body mass index, Eastern Cooperative Oncology Group performance status (PS), serum PSA level, blood count and blood biochemical data including AST and ALT, clinical T stage, metastatic sites, tumor burden according to CHAARTED criteria^25^, prior local therapy, time to castration resistance (TTCR), and first-line agent for CRPC were respectively collected and assessed. AST/ALT ratio was calculated for each patient four different times using serum AST and ALT; at PC diagnosis, PSA nadir during ADT treatment, CRPC diagnosis, and after CRPC treatment. For surviving patients, ‘after CRPC treatment’ was defined as the date of the final follow-up examination during CRPC treatment, while for deceased patients that was the date on which active treatment for CRPC was discontinued.

Bone scintigraphy, computerized tomography, and magnetic resonance imaging findings were used for staging at the time of PC diagnosis. During the treatment course, these imaging modalities were performed when necessary, such as when the disease progressed. Serum PSA was measured at intervals of 4–12 weeks depending on cancer status. CRPC was defined as serum testosterone level < 50 ng/dL, and either (1) PSA level measured at intervals of 4 weeks or more increased by ≥ 25% from the lowest value, with the increase ≥ 2.0 ng/mL, or (2) progression shown by imaging or appearance of new lesions^33^. TTCR was defined as duration from initiation of initial ADT treatment to first stated date of CRPC and OS as duration from date of diagnosis of PC to death during any course. The median follow-up duration from first PC diagnosis for the entire cohort was 57.2 months. Clinicopathologic features of all 130 CRPC patients are summarized in Table 1.

### Statistical analyses

Measurement values are expressed as median (interquartile range; IQR), mean ± standard deviation (SD), or number (percent of total). The Wilcoxon signed-rank test was used to compare AST/ALT ratio, AST, and ALT at PC diagnosis and three subsequent time points. Receiver-operating curve (ROC) and Youden’s index values for AST/ALT ratio for predicting lethality were used to determine optimum threshold. To analyze differences in patient characteristics after grouping with AST/ALT ratio, Student’s t-test or a Chi-square test was used, while Mann–Whitney’s U test was utilized to evaluate non-normal distributed continuous variables between groups. Survival curves were produced using the Kaplan–Meier method, with differences analyzed by a log-rank test. Uni- and multivariate analyses for OS and TTCR were performed using a Cox proportional hazards regression model. A p value less than 0.05 was considered to indicate statistical significance. All data were analyzed using the Easy R (EZR) statistical software application (http://www.jichi.ac.jp/saitama-sct/SaitamaHP.files/statmed.html)^34^.

### Supplementary Information

Supplementary Information 1.

Supplementary Information 2.

## Data Availability

The datasets generated and/or analyzed during the current study are available from the corresponding author on reasonable request.
