# Supplementary material for: Longitudinal change in castration-resistant prostate cancer biomarker AST/ALT ratio reflects tumor progression
Source: Sci Rep. 2023 Sep 15;13:15292. doi: 10.1038/s41598-023-42711-z (PMC10504303; doi:10.1038/s41598-023-42711-z)
Supplement: Supplementary file 2 — Supplementary Information 2. [file 41598_2023_42711_MOESM2_ESM.pptx]

## Slide 1
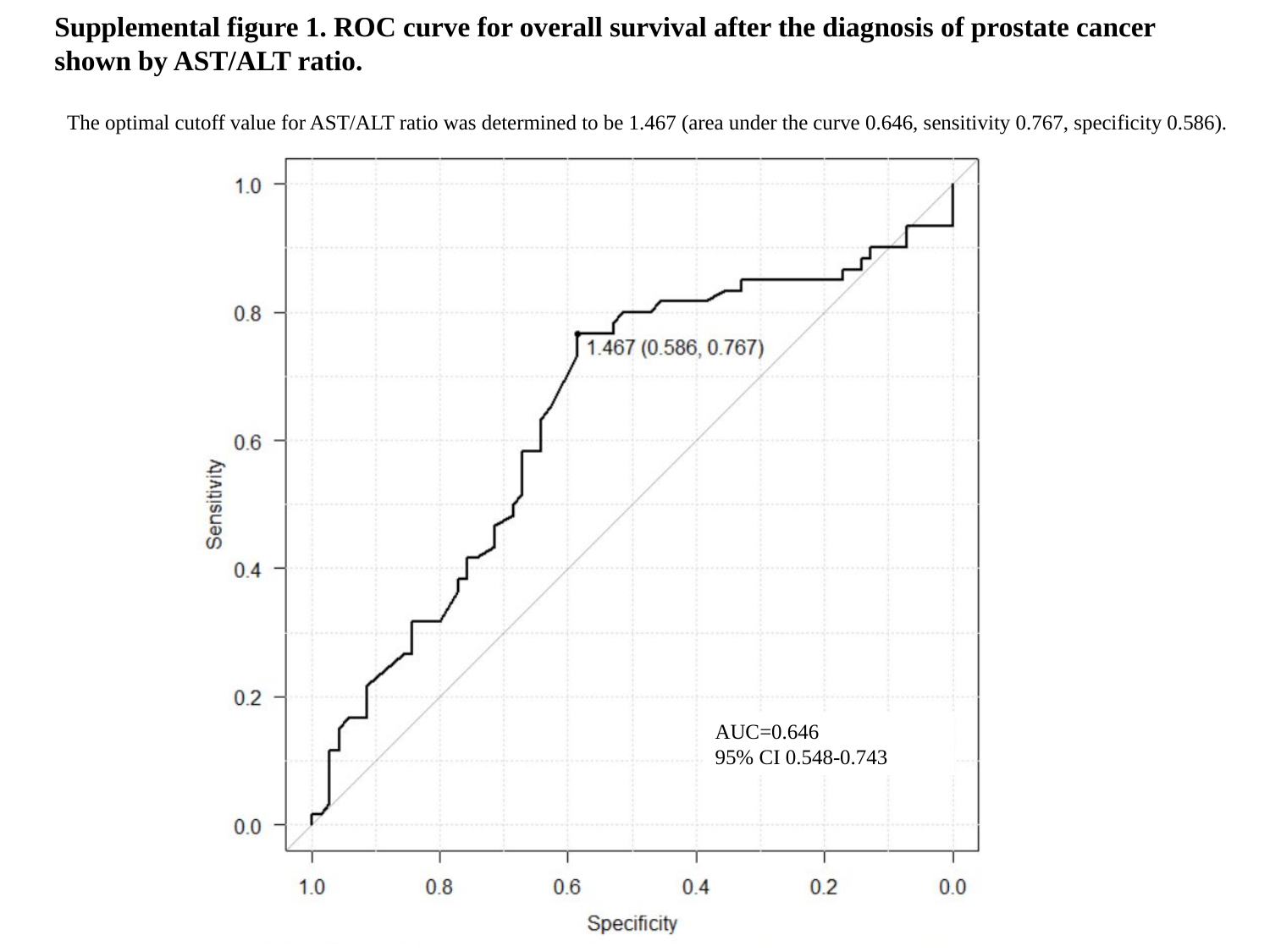

Supplemental figure 1. ROC curve for overall survival after the diagnosis of prostate cancer shown by AST/ALT ratio.
The optimal cutoff value for AST/ALT ratio was determined to be 1.467 (area under the curve 0.646, sensitivity 0.767, specificity 0.586).
AUC=0.646
95% CI 0.548-0.743
